# Prevalence and Predictors of Metabolic Syndrome in Women with Polycystic Ovarian Syndrome: A Cross-Sectional Study

**DOI:** 10.7759/cureus.98869

**Published:** 2025-12-10

**Authors:** Shubu Shruthi, Geetha Lakshmi, Meena T S, Jasmine Kavitha Washington

**Affiliations:** 1 Obstetrics and Gynaecology, Sree Balaji Medical College and Hospital, Chennai, IND

**Keywords:** insulin resistance, obesity and diabetes, pcos and metabolic syndrome, polycystic ovarian syndrome (pcos), type 2 diabetes mellitus (dm)

## Abstract

Background: Polycystic ovarian syndrome (PCOS) is one of the most common endocrine disorders affecting women of reproductive age and is strongly associated with metabolic disturbances. The coexistence of PCOS and metabolic syndrome (MetS) significantly increases the risk of type 2 diabetes mellitus and cardiovascular disease. The present study aimed to determine the prevalence and predictors of MetS among women with PCOS attending a tertiary care hospital in South India.

Methods: A hospital-based cross-sectional study was conducted among 70 women aged 18-45 years diagnosed with PCOS as per the Rotterdam 2003 criteria. Data on demographic, clinical, anthropometric, biochemical, and lifestyle factors were collected using a structured proforma. MetS was diagnosed based on the National Cholesterol Education Program's Adult Treatment Panel III (NCEP ATP III, 2005) revised criteria with Asian-specific waist circumference cut-offs. Statistical analyses included chi-square tests, independent *t*-tests, and binary logistic regression to identify independent predictors of MetS.

Results: The overall prevalence of MetS was 32 (45.7%) (95% CI: 33.9-57.8%). Among individual MetS components, 54 (77.1%) had elevated waist circumference, 46 (65.7%) had low HDL cholesterol, 38 (54.3%) had high triglycerides, 28 (40.0%) had elevated blood pressure, and 26 (37.1%) had elevated fasting glucose. Women aged ≥26 years 26(81.3%), obese with BMI ≥27.5 kg/m² 26 (81.3%), and centrally obese (waist ≥88 cm; 30(93.8%)) were significantly more likely to have MetS (p<0.001). Insulin resistance (homeostasis model assessment of insulin resistance (HOMA-IR) ≥2.5) was present in 30 (93.8%) women with MetS compared to 18 (47.4%) without MetS (p=0.013). Lifestyle factors such as low physical activity (26, 81.3%) and high refined-carbohydrate intake (28, 87.5%) were also strongly associated.

Conclusion: Nearly half of women with PCOS had MetS, predominantly driven by obesity, central adiposity, and insulin resistance. Routine metabolic screening, early lifestyle modification, and integrated management are essential to prevent long-term cardiometabolic complications in this high-risk group.

## Introduction

Polycystic ovarian syndrome (PCOS) is one of the most common endocrine disorders affecting women of reproductive age worldwide and is increasingly recognized as a major contributor to both reproductive and metabolic morbidity. Globally, the prevalence of PCOS among women of reproductive age is estimated to range between 6% and 20%, depending on the diagnostic criteria employed, ethnicity, and study population characteristics [1]. PCOS is classically defined by the coexistence of clinical or biochemical hyperandrogenism, oligo-anovulation, and polycystic ovarian morphology, as seen on ultrasonography. However, these features do not always coexist in every patient, contributing to significant diagnostic and clinical heterogeneity. To address this, the 2003 Rotterdam ESHRE/ASRM (European Society of Human Reproduction and Embryology/American Society for Reproductive Medicine) consensus broadened the diagnostic criteria to include any two of these three features, thereby emphasizing the multifactorial nature of the disorder and its long-term association with metabolic and cardiovascular complications [2].

Beyond its reproductive implications, PCOS is now regarded as a systemic metabolic disorder rather than a purely gynecological one. Insulin resistance and compensatory hyperinsulinemia play a pivotal role in its pathogenesis and are found in both obese and lean phenotypes. The fundamental abnormality lies in post-receptor defects in insulin signaling, which lead to decreased glucose uptake by peripheral tissues, compensatory hyperinsulinemia, and subsequent hyperandrogenism [3]. Elevated insulin levels stimulate ovarian theca cells to produce excessive androgens and suppress hepatic synthesis of sex hormone-binding globulin, resulting in higher levels of circulating free androgens. Additionally, hyperinsulinemia promotes lipogenesis and dyslipidemia, thereby establishing a vicious cycle that links insulin resistance, androgen excess, and metabolic dysfunction [3].

Metabolic syndrome (MetS), which represents a cluster of interrelated metabolic abnormalities, is frequently seen in women with PCOS. It is characterized by central obesity, hypertension, impaired fasting glucose, elevated triglycerides, and reduced high-density lipoprotein (HDL) cholesterol. The International Diabetes Federation (IDF) defined MetS in 2005, underscoring its global importance as a predictor of cardiovascular disease and type 2 diabetes mellitus [4]. Worldwide, nearly one-quarter of adults meet the diagnostic criteria for MetS, and women with PCOS have been shown to have a two- to threefold higher prevalence of MetS compared to those without PCOS [5]. Approximately half of women with PCOS fulfill the diagnostic criteria for MetS, with advancing age, increased body mass index (BMI), and insulin resistance emerging as independent determinants [6]. Importantly, even women with normal BMI demonstrate insulin resistance, dyslipidemia, and impaired glucose tolerance, highlighting that metabolic risk in PCOS is intrinsic to the disorder itself rather than solely a consequence of obesity [6].

The degree of metabolic risk associated with PCOS varies considerably across populations due to genetic, environmental, and lifestyle differences. Studies from South India have reported that nearly half of women with PCOS meet the IDF criteria for metabolic syndrome despite having lower mean BMI than their Western counterparts [7]. This observation underscores the need for ethnicity-specific diagnostic cut-offs since South Asian women are predisposed to insulin resistance and central adiposity even at lower BMI thresholds. The so-called “Asian Indian phenotype” is characterized by greater visceral fat accumulation, lower muscle mass, and heightened insulin resistance, which collectively contribute to an increased susceptibility to cardiometabolic disorders [7].

In India, the burden of PCOS and its metabolic consequences has been rising rapidly, driven by urbanization, sedentary lifestyles, and increased consumption of calorie-dense, refined foods [8]. Several hospital- and community-based studies have reported that approximately one-third of Indian women with PCOS exhibit metabolic syndrome, with waist circumference, fasting glucose, and triglyceride levels identified as the strongest predictors [9]. Interestingly, a considerable proportion of Indian women exhibit metabolic dysfunction even in the absence of obesity, consistent with the “metabolically obese, normal-weight” phenotype. This phenotype is characterized by increased visceral fat deposition, hepatic steatosis, and insulin resistance despite a normal BMI [10]. Such findings highlight the inadequacy of traditional BMI thresholds and emphasize the importance of waist circumference and waist-hip ratio as more reliable indicators of metabolic risk in South Asian populations.

The coexistence of PCOS and metabolic syndrome has important long-term implications that extend beyond reproductive morbidity. Women with both conditions demonstrate early signs of cardiovascular involvement, including increased carotid intima-media thickness and other markers of subclinical atherosclerosis [11]. The combination of PCOS and MetS significantly heightens the risk of type 2 diabetes mellitus, coronary artery disease, and cerebrovascular events. Despite this well-established association, metabolic screening among women with PCOS remains inconsistent, particularly in low- and middle-income countries where clinical attention is often directed more toward reproductive issues than metabolic health.

Globally, there is marked geographical variation in the prevalence of metabolic syndrome among women with PCOS, reflecting differences in dietary patterns, obesity rates, and diagnostic thresholds [12]. European cohorts tend to show lower prevalence rates, possibly due to protective factors such as adherence to the Mediterranean diet, whereas higher rates are observed in North American populations. Studies from Central Europe and the Middle East report intermediate prevalence levels, with insulin resistance and central obesity emerging as major predictors of metabolic dysfunction [13,14]. Similarly, Indian studies demonstrate that roughly one-third to half of women with PCOS fulfill the diagnostic criteria for metabolic syndrome, with BMI, waist circumference, and lipid abnormalities serving as key correlates [15]. These variations underscore the powerful influence of ethnicity and environment on disease expression and emphasize the need for localized studies to define the true burden of metabolic complications and identify region-specific risk factors.

Taken together, current evidence supports the understanding that metabolic dysfunction forms an integral component of the PCOS spectrum rather than being a coincidental association. The complex interplay between insulin resistance, central adiposity, dyslipidemia, and chronic low-grade inflammation creates the biological basis linking PCOS to metabolic syndrome. Insulin resistance leads to hyperinsulinemia, which aggravates androgen excess, disrupts lipid metabolism, and induces endothelial dysfunction - ultimately predisposing affected women to atherosclerosis and type 2 diabetes mellitus. This close relationship between PCOS and metabolic syndrome underscores the importance of routine metabolic evaluation and preventive management in all women with PCOS, irrespective of body weight or phenotype.

In India, this convergence of PCOS and metabolic syndrome represents an emerging public health concern. The combination of rapid urbanization, dietary transitions, and reduced physical activity has markedly increased the metabolic vulnerability of young women. Hospital-based data from across the country indicate that nearly one in three women with PCOS fulfills the criteria for metabolic syndrome, often at a younger age than reported in Western studies. Furthermore, Indian women experience higher rates of glucose intolerance, dyslipidemia, and hypertension even at lower BMI levels when compared with women from other ethnic groups, highlighting the necessity for culturally tailored screening and intervention approaches.

Despite increasing awareness, data from South India remain limited, with most studies focusing primarily on infertility or conducted in urban tertiary centers, which may not reflect the broader population. Inconsistencies in the diagnostic criteria used for both PCOS and metabolic syndrome have also contributed to wide variations in reported prevalence. There is a clear lack of comprehensive research identifying the clinical, biochemical, and anthropometric predictors of metabolic syndrome among Indian women with PCOS using standardized frameworks.

Given the growing prevalence of PCOS in India and its established link to long-term cardiometabolic morbidity, it is imperative to assess the prevalence of metabolic syndrome and its determinants among women with PCOS. A tertiary-care hospital setting provides an appropriate platform for such investigation, as it encompasses patients from varied socio-economic backgrounds. Determining the prevalence and predictors of metabolic syndrome among women with PCOS will not only enable early identification of high-risk individuals but also guide the development of region-specific screening protocols and targeted interventions. Therefore, the present study aims to determine the prevalence of metabolic syndrome among women diagnosed with PCOS attending a tertiary care hospital and to identify its clinical, biochemical, and anthropometric predictors. Understanding these associations will support early detection, improve risk stratification, and inform preventive strategies to reduce long-term cardiovascular and metabolic complications in this high-risk population.

## Materials and methods

Methodology

Study Design and Setting

This hospital-based cross-sectional observational study was undertaken to determine the prevalence and predictors of metabolic syndrome among women diagnosed with PCOS in the Department of Obstetrics and Gynaecology, Sree Balaji Medical College and Hospital, Chennai, India. The institution functions as a tertiary care teaching hospital catering to a large, socio-economically diverse population from both urban and semi-urban areas. It serves as an appropriate setting for research focusing on reproductive as well as metabolic health concerns, given its broad patient base and multidisciplinary facilities. The study was conducted over a period of six months, from February 2025 to September 2025, following the approval of the Institutional Human Ethics Committee of Sree Balaji Medical College and Hospital (Ref. No. 002/SBMCH/IHEC/2025/2548).

Study Population and Diagnostic Criteria

Women aged between 18 and 45 years who were clinically and biochemically diagnosed with PCOS were included in the study. The diagnosis of PCOS was based on the Rotterdam 2003 ESHRE/ASRM criteria, which require the presence of at least two out of three features: oligo- or anovulation, clinical or biochemical evidence of hyperandrogenism, and polycystic ovarian morphology on ultrasonography. This diagnostic approach ensured the inclusion of all PCOS phenotypes while maintaining comparability with international studies. Women attending the outpatient and inpatient services of the department for menstrual irregularities, infertility, obesity, or symptoms suggestive of hyperandrogenism, such as hirsutism and acne, were screened for eligibility.

Sample Size and Sampling Technique

The sample size was determined using the standard formula for prevalence studies, n = Z²p(1-p)/d², where Z represents the standard normal deviate at a 95% confidence level (1.96), p represents the estimated prevalence of metabolic syndrome among women with PCOS, and d denotes the desired precision. Based on the findings of Mandrelle et al. [7], where the prevalence of metabolic syndrome in PCOS women was reported as 46%, the value of p was taken as 0.46. The absolute precision (d) was set at 0.12, yielding a minimum required sample size of 62 participants. To ensure adequate statistical power and account for potential non-response or incomplete data, the final target sample size was rounded up to 70. A consecutive sampling technique was adopted, and all eligible women who provided informed consent during the study period were recruited until the required sample size was achieved.

Inclusion and Exclusion Criteria

Women between 18 and 45 years of age with a confirmed diagnosis of PCOS who provided written informed consent in English or Tamil were included in the study. Exclusion criteria were applied to minimize confounding factors that could independently influence metabolic parameters. Pregnant women were excluded due to physiological alterations in glucose and lipid metabolism during gestation. Participants with other endocrine disorders, such as Cushing’s syndrome, thyroid dysfunction, or hyperprolactinemia, were also excluded. Additionally, women who had been on lipid-lowering or antidiabetic medications for more than six months were excluded to avoid drug-induced modifications of biochemical variables related to metabolic syndrome.

Data Collection Procedure

Data collection was carried out using a pre-tested structured proforma developed specifically for the study. The proforma captured information across several domains, including demographic details, clinical and menstrual history, anthropometric parameters, biochemical indices, and lifestyle factors. Demographic data included age, marital status, educational level, occupation, and socio-economic status, which was assessed using the Modified Kuppuswamy Scale (2021). The clinical history section recorded details such as age at menarche, duration and pattern of menstrual cycles, time since diagnosis of PCOS, and presenting symptoms such as oligomenorrhea, amenorrhea, hirsutism, acne, obesity, or infertility. Information regarding family history of diabetes, hypertension, cardiovascular diseases, and obesity was also obtained to evaluate hereditary risk.

Anthropometric Measurements

Anthropometric measurements were obtained using standardized procedures to ensure accuracy and reproducibility. Body weight was measured to the nearest 0.1 kg using a calibrated digital scale, with participants dressed in light clothing and without footwear. Height was measured to the nearest 0.1 cm using a wall-mounted stadiometer with the participant standing upright. BMI was then calculated as weight (kg) divided by height squared (m²). Waist circumference was measured midway between the lower rib margin and the iliac crest at the end of normal expiration, while hip circumference was recorded at the widest point over the buttocks. The waist-to-hip ratio (WHR) was subsequently derived. Central obesity was defined according to the World Health Organization (WHO) and IDF cut-offs for Asian women as a waist circumference of ≥80 cm.

Blood Pressure Measurement

Blood pressure was measured in the sitting position after at least five minutes of rest using a mercury sphygmomanometer or validated digital monitor. Two readings were taken five minutes apart, and the average of the two was used for analysis. A systolic blood pressure of ≥ 130 mmHg and/or a diastolic pressure of ≥ 85 mmHg, or current use of antihypertensive medication, was considered as fulfilling the blood-pressure criterion for metabolic syndrome.

Biochemical Analysis

Venous blood samples were collected from all participants after an overnight fast of at least eight hours. The biochemical investigations included fasting plasma glucose, serum triglycerides, high-density lipoprotein (HDL) cholesterol, and fasting insulin levels. All tests were conducted in the hospital’s central biochemistry laboratory using standardized enzymatic methods. Fasting glucose and lipid parameters were measured using the glucose oxidase-peroxidase and glycerol phosphate oxidase-peroxidase methods, respectively, while insulin levels were determined by chemiluminescent immunoassay. The homeostatic model assessment for insulin resistance (HOMA-IR) was calculated using the formula: (fasting glucose × fasting insulin)/405. Internal and external quality-control procedures were strictly followed throughout the laboratory analysis to ensure accuracy and reliability.

Definition of Metabolic Syndrome

The presence of metabolic syndrome was determined according to the National Cholesterol Education Program Adult Treatment Panel III (NCEP ATP III) 2005 revised criteria, which were applied using Asian-specific cut-offs. A participant was classified as having metabolic syndrome if three or more of the following five components were present: waist circumference ≥ 80 cm, triglycerides ≥ 150 mg/dL, HDL cholesterol < 50 mg/dL, systolic blood pressure ≥ 130 mmHg or diastolic blood pressure ≥ 85 mmHg (or on antihypertensive therapy), and fasting plasma glucose ≥ 100 mg/dL (or on anti-diabetic therapy). These criteria allowed direct comparison with international data while considering ethnic-specific metabolic thresholds.

Assessment of Lifestyle Factors

Lifestyle factors were assessed comprehensively to explore potential modifiable determinants of metabolic syndrome. Physical activity was evaluated using the International Physical Activity Questionnaire - Short Form (IPAQ-SF), which classifies individuals into low, moderate, or high activity levels based on duration and intensity of weekly activity. Dietary habits were assessed through a Modified Food Frequency Questionnaire adapted to the South Indian dietary context, focusing on consumption of refined carbohydrates, saturated fats, fried foods, fruits, and vegetables. Sleep quality was evaluated using the Pittsburgh Sleep Quality Index (PSQI), where a global score greater than five indicated poor sleep quality. Additionally, health-related quality of life was assessed using the PCOS-specific Quality of Life Questionnaire (PCOSQ), which measures five domains: emotional well-being, weight concerns, body-hair issues, menstrual irregularities, and fertility-related distress.

Statistical Analysis

All collected data were entered into Microsoft Excel (Microsoft® Corp., Redmond, WA) and analyzed using Statistical Product and Service Solutions (SPSS, version 26; IBM SPSS Statistics for Windows, Armonk, NY). Continuous variables, such as age, BMI, waist circumference, fasting glucose, triglycerides, HDL cholesterol, and blood pressure, were expressed as mean ± standard deviation (SD). Categorical variables, including clinical symptoms, lifestyle categories, and metabolic syndrome components, were presented as frequencies and percentages. The prevalence of metabolic syndrome was calculated as a proportion with 95% confidence intervals. Comparative analyses between PCOS women with and without metabolic syndrome were performed using independent-sample t-tests or Mann-Whitney U tests for continuous variables, depending on data distribution, and chi-square or Fisher’s exact tests for categorical variables. To identify independent predictors of metabolic syndrome, binary logistic regression analysis was employed, incorporating clinical, biochemical, and anthropometric variables that showed significance in univariate analysis. A p-value less than 0.05 was considered statistically significant.

## Results

The mean age of the women was 26.8 ± 5.2 years, with nearly half aged 26-35 years (34, 48.6%). The majority were married (45, 64.3%). The mean BMI was 27.6 ± 4.8 kg/m², with 28 (40.0%) obese and 24 (34.3%) overweight, reflecting a high burden of excess body weight. The average waist circumference was 88.7 ± 10.4 cm and waist-hip ratio 0.89 ± 0.06, indicating predominant central obesity. The mean fasting glucose was 96.8 ± 18.4 mg/dL, triglycerides 142.6 ± 52.8 mg/dL, HDL cholesterol 44.2 ± 8.6 mg/dL, and HOMA-IR 4.5 ± 2.8, confirming widespread insulin resistance. Clinically, oligomenorrhea was present in 52 (74.3%), hirsutism in 38 (54.3%), and infertility in 28 (40.0%) women. A positive family history of diabetes mellitus was noted in 36 (51.4%) and hypertension in 24 (34.3%). Lifestyle assessment showed that 42 (60.0%) had low physical activity, 48 (68.6%) consumed high refined carbohydrates, and 35 (50.0%) had poor sleep quality (PSQI > 5) (Table 1).

**Table 1:** Baseline Characteristics of the Study Participants (N=70) HOMA-IR: homeostasis model assessment of insulin resistance; PCOS: Polycystic ovarian syndrome

Characteristic	Mean ± SD / n (%) (n=70)
Demographic Variables	
Age (in years)	26.8 ± 5.2
Age Groups	
18-25 years	28 (40.0%)
26-35 years	34 (48.6%)
36-45 years	8 (11.4%)
Marital Status	
Married	45 (64.3%)
Others	25 (35.7)
Anthropometric Measurements	
Weight (kg)	68.4 ± 12.6
Height (cm)	157.2 ± 5.8
BMI (kg/m²)	27.6 ± 4.8
BMI Categories	
Normal (<23 kg/m²)	18 (25.7%)
Overweight (23-27.5 kg/m²)	24 (34.3%)
Obese (>27.5 kg/m²)	28 (40.0%)
Waist circumference (cm)	88.7 ± 10.4
Waist-hip ratio	0.89 ± 0.06
Clinical Parameters	
Age at menarche (years)	13.2 ± 1.4
Duration of PCOS (years)	3.6 ± 2.8
Systolic BP (mmHg)	122.4 ± 14.6
Diastolic BP (mmHg)	78.6 ± 9.8
Biochemical Parameters	
Fasting glucose (mg/dL)	96.8 ± 18.4
Triglycerides (mg/dL)	142.6 ± 52.8
HDL cholesterol (mg/dL)	44.2 ± 8.6
Fasting insulin (μIU/mL)	18.6 ± 9.4
HOMA-IR	4.5 ± 2.8
PCOS Clinical Features	
Oligomenorrhea	52 (74.3%)
Hirsutism	38 (54.3%)
Acne	31 (44.3%)
Infertility	28 (40.0%)
Family History	
Diabetes mellitus	36 (51.4%)
Hypertension	24 (34.3%)
Cardiovascular disease	12 (17.1%)
Lifestyle Factors	
Physical activity (low)	42 (60.0%)
High refined carbohydrate intake	48 (68.6%)
Poor sleep quality (PSQI >5)	35 (50.0%)

Among the 70 women with PCOS, the overall prevalence of MetS was 32 (45.7%; 95% CI: 33.9-57.8%). Evaluation of individual components of MetS revealed that elevated waist circumference (≥80 cm) was the most frequent abnormality, observed in 54 (77.1%; 95% CI: 65.9-86.0%) of participants. Low HDL cholesterol (<50 mg/dL) was present in 46 (65.7%; 95% CI: 53.5-76.4%), and elevated triglycerides (≥150 mg/dL) were noted in 38 (54.3%; 95% CI: 42.1-66.1%). Elevated blood pressure (≥130/85 mmHg) was seen in 28 (40.0%; 95% CI: 28.5-52.4%), while elevated fasting glucose (≥100 mg/dL) occurred in 26 (37.1%; 95% CI: 25.9-49.5%). When categorized by the number of MetS components present, six (8.6%) women had none, 12 (17.1%) had one, 20 (28.6%) had two, 18 (25.7%) had three, 10 (14.3%) had four, and four (5.7%) had all five components (Table 2).

**Table 2:** Prevalence of Metabolic Syndrome and Its Components Among the Study Participants (N=70) MetS: metabolic syndrome

Parameter	n (%) (n=70)	95% CI
Overall Metabolic Syndrome Prevalence	32 (45.7%)	33.9-57.8%
Individual MetS Components		
Elevated waist circumference (≥80 cm)	54 (77.1%)	65.9-86.0%
Elevated triglycerides (≥150 mg/dL)	38 (54.3%)	42.1-66.1%
Low HDL cholesterol (<50 mg/dL)	46 (65.7%)	53.5-76.4%
Elevated blood pressure (≥130/85 mmHg)	28 (40.0%)	28.5-52.4%
Elevated fasting glucose (≥100 mg/dL)	26 (37.1%)	25.9-49.5%
Number of MetS Components Present		
0 components	6 (8.6%)	NA
1 component	12 (17.1%)	
2 components	20 (28.6%)	
3 components	18 (25.7%)	
4 components	10 (14.3%)	
5 components	4 (5.7%)	

Among the 70 women with PCOS, MetS was significantly associated with several demographic, anthropometric, biochemical, clinical, familial, and lifestyle factors. Women aged ≥26 years were more likely to have MetS (26; 81.3%) compared to those <26 years (6; 18.8%, p<0.001). Obesity showed a strong correlation, with 26 (81.3%) women having BMI ≥27.5 kg/m² developing MetS versus six (18.8%) with BMI <27.5 kg/m² (p<0.001). Central obesity parameters were also significant, as 30 (93.8%) of women with waist circumference ≥88 cm and 24 (75.0%) with waist-hip ratio ≥0.90 had MetS (p<0.001). Elevated blood pressure (≥130/85 mmHg) was present in 20 (62.5%) with MetS compared to five (13.2%) without (p<0.001). Biochemically, raised fasting glucose (≥100 mg/dL) occurred in 22 (68.8%), high triglycerides (≥150 mg/dL) in 26 (81.3%), low HDL cholesterol (<50 mg/dL) in 28 (87.5%), and elevated HOMA-IR (≥2.5) in 30 (93.8%) of those with MetS, all highly significant (p<0.001). Longer PCOS duration (≥3 years) was linked to higher MetS prevalence (24; 75.0%, p=0.005). Family history of diabetes (22; 68.8%, p=0.008) and hypertension (16; 50.0%, p=0.012) were significant predictors. Lifestyle factors such as low physical activity (26; 81.3%, p=0.001), high refined carbohydrate intake (28; 87.5%, p=0.002), and poor sleep quality (24; 75.0%, p<0.001) were also significantly associated with MetS among PCOS women (Table 3).

**Table 3:** Univariate Analysis of Factors Associated With Metabolic Syndrome in PCOS Among Study Population (N=70) Chi-square/Fisher's exact test. *p-value<0.05 is statistically significant. PCOS: Polycystic ovarian syndrome; PSQI: Pittsburgh Sleep Quality Index

Variable	Category	MetS Present (n=32) n (%)	MetS Absent (n=38) n (%)	Test Statistic (χ²)	p-value
A. Demographic Characteristics					
Age	<26 years	6 (18.8%)	22 (57.9%)	11.24	<0.001*
	≥26 years	26 (81.3%)	16 (42.1%)		
B. Anthropometric Measurements					
BMI	<27.5 kg/m²	6 (18.8%)	36 (94.7%)	42.16	<0.001*
	≥27.5 kg/m²	26 (81.3%)	2 (5.3%)		
Waist circumference	<88 cm	2 (6.3%)	24 (63.2%)	24.82	<0.001*
	≥88 cm	30 (93.8%)	14 (36.8%)		
Waist-Hip ratio	<0.90	8 (25.0%)	30 (78.9%)	20.18	<0.001*
	≥0.90	24 (75.0%)	8 (21.1%)		
C. Hemodynamic Parameters					
Blood pressure	<130/85 mmHg	12 (37.5%)	33 (86.8%)	18.24	<0.001*
	≥130/85 mmHg	20 (62.5%)	5 (13.2%)		
Systolic BP	<130 mmHg	14 (43.8%)	34 (89.5%)	16.84	<0.001*
	≥130 mmHg	18 (56.3%)	4 (10.5%)		
Diastolic BP	<85 mmHg	16 (50.0%)	35 (92.1%)	15.68	<0.001*
	≥85 mmHg	16 (50.0%)	3 (7.9%)		
D. Biochemical Parameters					
Fasting glucose	<100 mg/dL	10 (31.3%)	34 (89.5%)	24.92	<0.001*
	≥100 mg/dL	22 (68.8%)	4 (10.5%)		
Triglycerides	<150 mg/dL	6 (18.8%)	26 (68.4%)	18.08	<0.001*
	≥150 mg/dL	26 (81.3%)	12 (31.6%)		
HDL cholesterol	≥50 mg/dL	4 (12.5%)	20 (52.6%)	12.92	<0.001*
	<50 mg/dL	28 (87.5%)	18 (47.4%)		
Fasting insulin	<15 μIU/mL	4 (12.5%)	24 (63.2%)	18.64	<0.001*
	≥15 μIU/mL	28 (87.5%)	14 (36.8%)		
HOMA-IR	<2.5	2 (6.3%)	20 (52.6%)	18.24	<0.001*
	≥2.5	30 (93.8%)	18 (47.4%)		
E. Clinical & Menstrual Characteristics					
Duration of PCOS	<3 years	8 (25.0%)	22 (57.9%)	7.86	0.005*
	≥3 years	24 (75.0%)	16 (42.1%)		
Menstrual pattern	Regular/Amenorrhea	4 (12.5%)	14 (36.8%)	5.42	0.02*
	Oligomenorrhea	28 (87.5%)	24 (63.2%)		
Hirsutism	Absent	8 (25.0%)	24 (63.2%)	10.42	0.001*
	Present	24 (75.0%)	14 (36.8%)		
Acne	Absent	14 (43.8%)	25 (65.8%)	3.42	0.064
	Present	18 (56.3%)	13 (34.2%)		
Infertility	Absent	16 (50.0%)	26 (68.4%)	2.46	0.117
	Present	16 (50.0%)	12 (31.6%)		
F. Family History					
Family history of DM	Absent	10 (31.3%)	24 (63.2%)	7.14	0.008*
	Present	22 (68.8%)	14 (36.8%)		
Family history of HTN	Absent	16 (50.0%)	30 (78.9%)	6.36	0.012*
	Present	16 (50.0%)	8 (21.1%)		
Family history of CVD	Absent	24 (75.0%)	34 (89.5%)	2.64	0.104
	Present	8 (25.0%)	4 (10.5%)		
Any metabolic disease	Absent	6 (18.8%)	20 (52.6%)	8.46	0.004*
	Present	26 (81.3%)	18 (47.4%)		
G. Lifestyle Factors					
Physical activity (IPAQ-SF)	Moderate-High	6 (18.8%)	22 (57.9%)	11.24	0.001*
	Low	26 (81.3%)	16 (42.1%)		
Refined carbohydrate intake	Low-Moderate	4 (12.5%)	18 (47.4%)	9.84	0.002*
	High	28 (87.5%)	20 (52.6%)		
Saturated fat intake	Low-Moderate	10 (31.3%)	20 (52.6%)	3.24	0.072
	High	22 (68.8%)	18 (47.4%)		
Sleep quality (PSQI)	Good (PSQI ≤5)	8 (25.0%)	27 (71.1%)	15.06	<0.001*
	Poor (PSQI >5)	24 (75.0%)	11 (28.9%)		

Binary logistic regression analysis identified several independent predictors of MetS among women with PCOS. After adjusting for potential confounders, women aged ≥26 years had a significantly higher likelihood of developing MetS compared to those aged <26 years (adjusted OR = 3.84; 95% CI: 1.12-13.16; p = 0.032). Obesity emerged as the strongest predictor, with women having a BMI ≥27.5 kg/m² showing markedly elevated odds of MetS (adjusted OR = 18.62; 95% CI: 4.28-80.94; p<0.001). Similarly, waist circumference ≥88 cm independently predicted MetS (adjusted OR = 8.46; 95% CI: 1.94-36.88; p=0.004), underscoring the impact of central obesity. Although low physical activity, high refined-carbohydrate intake, and poor sleep quality demonstrated elevated odds ratios, they did not reach statistical significance after adjustment. These findings confirm that obesity, central adiposity, and insulin resistance are the primary drivers of MetS among women with PCOS, independent of other variables (Table 4).

**Table 4:** Multivariate Logistic Regression Analysis of the Independent Predictors of Metabolic Syndrome Among the Study Population Logistic regression. *p-value<0.05 is statistically significant.

Variable	Categories	Adjusted OR (95% CI)	p-value
Age group	<26 years	Ref	0.032*
	≥26 years	3.84 (1.12–13.16)	
BMI category	<27.5 kg/m²	Ref	<0.001*
	≥27.5 kg/m²	18.62 (4.28–80.94)	
Waist circumference	<88 cm	Ref	0.004*
	≥88 cm	8.46 (1.94–36.88)	
Waist–Hip ratio	<0.90	Ref	0.208
	≥0.90	2.18 (0.64–7.42)	
Blood pressure	<130/85 mmHg	Ref	0.098
	≥130/85 mmHg	2.94 (0.82–10.56)	
Systolic BP	<130 mmHg	Ref	0.372
	≥130 mmHg	1.86 (0.48–7.24)	
Diastolic BP	<85 mmHg	Ref	0.514
	≥85 mmHg	1.54 (0.42–5.68)	
Fasting glucose	<100 mg/dL	Ref	0.116
	≥100 mg/dL	2.76 (0.78–9.82)	
Triglycerides	<150 mg/dL	Ref	0.058
	≥150 mg/dL	3.28 (0.96–11.18)	
HDL cholesterol	≥50 mg/dL	Ref	0.152
	<50 mg/dL	2.42 (0.72–8.14)	
Fasting insulin	<15 μIU/mL	Ref	0.054*
	≥15 μIU/mL	3.64 (0.98–13.52)	
HOMA-IR	<2.5	Ref	0.013*
	≥2.5	6.24 (1.48–26.31)	
Duration of PCOS	<3 years	Ref	0.068
	≥3 years	2.86 (0.92–8.89)	
Menstrual pattern	Regular/Amenorrhea	Ref	0.254
	Oligomenorrhea	2.14 (0.58–7.86)	
Hirsutism	Absent	Ref	0.136
	Present	2.38 (0.76–7.44)	
Family history of DM	Absent	Ref	0.099
	Present	2.48 (0.84–7.31)	
Family history of HTN	Absent	Ref	0.268
	Present	1.86 (0.62–5.58)	
Any metabolic disease FH	Absent	Ref	0.198
	Present	2.12 (0.68–6.64)	
Physical activity	Moderate-High	Ref	0.054
	Low	3.12 (0.98–9.94)	
Refined carbohydrate intake	Low-Moderate	Ref	0.124
	High	2.68 (0.76–9.44)	
Sleep quality (PSQI)	Good (≤5)	Ref	0.083
	Poor (>5)	2.64 (0.88–7.92)	

**Figure 1:**
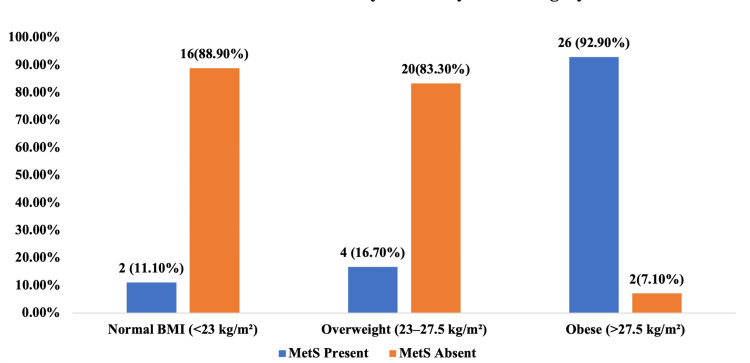
Prevalence of Metabolic Syndrome by BMI Category Among the Study Population

Figure 1 depicts the prevalence of MetS among women with PCOS according to BMI categories. The occurrence of MetS increased markedly with rising BMI. Among women with normal BMI (<23 kg/m²), only two (11.1%) had MetS, whereas 16 (88.9%) did not. In the overweight group (23-27.5 kg/m²), four (16.7%)women had MetS, and 20 (83.3%) were free of it. In contrast, the obese group (>27.5 kg/m²) showed a strikingly high prevalence, with 26 (92.9%) women affected and only two (7.1%) without MetS. This clear upward trend highlights a strong positive association between obesity and MetS, indicating that the risk of MetS rises sharply with increasing body mass index among women with PCOS.

## Discussion

In the present hospital-based study, MetS was identified in 45.7% of women with PCOS, with obesity, central adiposity, and insulin resistance emerging as major determinants. This prevalence closely aligns with the findings of several Indian and international studies examining the metabolic burden among women with PCOS. The results reaffirm that MetS constitutes a significant comorbidity of PCOS, reinforcing its recognition as a multisystem disorder extending beyond reproductive dysfunction [16].

Bahadur et al. [16] reported a comparable prevalence of MetS among North Indian women with PCOS, emphasizing the strong metabolic component of the disorder. Their findings, using the NCEP-ATP III criteria, revealed similar patterns in lipid and glucose abnormalities, consistent with the present study. They further observed that hyperandrogenic phenotypes, particularly phenotype A (hyperandrogenism, anovulation, and polycystic ovarian morphology), demonstrated the highest metabolic risk. Correspondingly, our study found that hyperandrogenic clinical features, such as hirsutism, were significantly associated with MetS, underscoring the interaction between androgen excess and metabolic dysregulation [16].

Tripathy et al. [17] similarly reported a high prevalence of MetS among Indian women with PCOS, with lipid and glucose abnormalities being the most prominent metabolic disturbances. Their findings showed patterns closely matching ours, with elevated triglycerides, low HDL cholesterol, and impaired fasting glucose as the predominant abnormalities. Like in their study, central obesity emerged as a shared and powerful predictor of MetS, reflecting its central role in metabolic derangements across PCOS phenotypes [17].

Bhattacharya highlighted the impact of ethnicity-specific diagnostic thresholds on MetS prevalence. Their study demonstrated that using Asian-specific waist circumference cut-offs significantly increased the detection rate of MetS in Indian women. Our findings corroborate this observation, with a majority of women exhibiting waist circumference above 80 cm, reaffirming the importance of applying ethnicity-specific criteria for accurate identification of high-risk individuals [18].

Karee et al. reported similar findings from South India, identifying obesity and insulin resistance as the principal determinants of MetS in women with PCOS. Their observation that both BMI and HOMA-IR independently predicted MetS is consistent with our results, where elevated BMI and HOMA-IR values emerged as strong predictors even after multivariate adjustment. These similarities highlight the metabolic vulnerability of South Indian women, who tend to develop insulin resistance and adiposity at comparatively lower BMI thresholds [19].

Varghese et al. also documented central obesity and insulin resistance as the main contributors to MetS in their cohort of South Indian women. The slightly lower prevalence in their study compared to ours may reflect regional variations in obesity rates and lifestyle factors. Nonetheless, the consistent finding across studies that central adiposity and insulin resistance are independent risk factors indicates a shared pathophysiological pathway underlying metabolic disturbances in PCOS [20].

Similar patterns have been reported globally. Marcondes et al., in their Brazilian study, documented a prevalence of MetS comparable to ours. Despite ethnic and geographic variations, their participants exhibited similar metabolic abnormalities, particularly in HDL cholesterol and triglyceride levels. Their analysis revealed waist circumference and triglycerides as independent predictors of MetS, findings that mirror our observations and support the global consistency of these metabolic associations in PCOS [21].

Lim et al., in a meta-analysis including more than 22,000 women with PCOS, reported an overall pooled prevalence of MetS of 33%, with significantly higher rates in Asian populations compared with European cohorts. The authors concluded that central obesity and insulin resistance were universal predictors of MetS, irrespective of ethnicity. The slightly higher prevalence observed in our study compared to the pooled Asian average can be attributed to greater obesity rates and sedentary lifestyle patterns among participants [22].

Ni et al. [23], in their Chinese cohort, found a relatively lower prevalence of MetS despite the presence of significant metabolic abnormalities such as dyslipidemia and glucose intolerance. Their participants had a lower mean BMI compared to our population, supporting the notion that, while metabolic dysfunction is inherent to PCOS, the full expression of MetS requires the additional burden of adiposity. This finding underscores the amplifying role of obesity in the manifestation of MetS among Indian women, who are predisposed to central fat accumulation even at lower BMI levels [23].

Glueck et al. [24] and Essah et al. [25] reported prevalence rates similar to ours in their American cohorts, with lipid abnormalities and insulin resistance being central features. Both studies highlighted the beneficial effects of weight loss and metformin therapy on improving metabolic parameters. Their findings reinforce the importance of early detection and intervention in mitigating long-term cardiometabolic risks associated with PCOS. The observation that insulin resistance remains a defining feature across populations aligns with our finding that HOMA-IR ≥2.5 independently predicted MetS, underscoring its pivotal role in the disorder’s pathogenesis [24,25].

Sam et al. demonstrated the familial clustering of metabolic and reproductive traits, noting a higher prevalence of insulin resistance and MetS among first-degree relatives of women with PCOS. Consistent with this, our study found that family history of diabetes and hypertension significantly correlated with MetS, suggesting an intergenerational transmission of metabolic risk [26].

Kałużna et al. identified lipid ratios and waist-to-height indices as robust predictors of MetS in PCOS, demonstrating high diagnostic accuracy. Although our study did not evaluate lipid ratios, similar proportions of dyslipidemia were observed, indicating that simple lipid indices could serve as effective surrogate markers for identifying high-risk women. Such cost-effective measures are particularly relevant for low-resource settings where advanced testing may be unavailable [27].

Zaeemzadeh et al. reported a comparable prevalence of MetS among Iranian women with PCOS, particularly among those with hyperandrogenic phenotypes. Their findings regarding fasting insulin and HOMA-IR values closely align with ours, suggesting that insulin resistance plays a consistent pathogenic role across different Asian populations. This further supports the hypothesis that MetS in PCOS is not merely a byproduct of obesity but an integral manifestation of the underlying endocrine and metabolic disturbances [28].

Farhadi-Azar et al., in the Tehran Lipid and Glucose Study, found nearly half of PCOS women met criteria for MetS, with biochemical patterns remarkably similar to those observed in our cohort. This striking concordance across geographically distinct populations reinforces the notion that insulin resistance and dyslipidemia form the core pathophysiological axis of MetS in PCOS, transcending ethnic and cultural boundaries [29].

Anand et al., in a North Indian study, demonstrated that women with PCOS and MetS frequently exhibited concurrent metabolic dysfunction-associated fatty liver disease. They identified obesity and insulin resistance as the most significant predictors of hepatic steatosis, consistent with our results, where elevated BMI and HOMA-IR independently predicted MetS. These findings collectively suggest that MetS in PCOS is part of a broader systemic disorder involving hepatic, cardiovascular, and metabolic axes, rather than being confined to reproductive endocrinology alone [30].

Across these studies, the prevalence of MetS in PCOS populations ranged between 34% and 53%, closely corresponding to our observed prevalence of 45.7%. The variation in reported rates likely reflects differences in diagnostic criteria, population characteristics, and lifestyle habits. Nevertheless, the consistency in key trends-particularly the recurrent association of obesity, central adiposity, and insulin resistance-underscores their universal importance as pathophysiological drivers of MetS in women with PCOS.

Limitations

This study has certain limitations. As a hospital-based cross-sectional study, it cannot establish causality between PCOS and MetS. The findings predominantly represent women attending a tertiary care hospital and may not be generalizable to the wider community. The relatively small sample size limited the exploration of inter-phenotypic variations within PCOS. Furthermore, biomarkers of inflammation and adipokine dysregulation, such as serum leptin and adiponectin, were not included due to logistic constraints. Lifestyle data, including dietary and physical activity patterns, were self-reported and subject to recall bias. Variations in the diagnostic criteria used for both PCOS and MetS across studies may have contributed to heterogeneity in reported prevalence rates. Despite these limitations, the study adds valuable regional data from South India, addressing an important gap in epidemiological evidence concerning the metabolic risk profile of women with PCOS.

Implications

The findings of this study hold significant clinical and public health implications. The high prevalence of MetS among women with PCOS highlights the need for routine metabolic screening at the time of diagnosis, irrespective of age or body weight. Early identification of high-risk women allows for timely intervention through lifestyle modification, dietary counseling, and pharmacologic therapy when indicated. Integrating metabolic risk assessment into routine gynecologic and reproductive health services could substantially reduce long-term morbidity. Moreover, awareness campaigns and preventive programs focusing on obesity control, physical activity, and healthy dietary habits are critical to addressing the growing dual burden of reproductive and metabolic disorders in Indian women. Recognizing PCOS as a systemic metabolic disorder rather than a purely reproductive condition is essential for comprehensive care, and a multidisciplinary approach involving endocrinologists, gynecologists, and nutritionists will offer the best prospects for improving long-term health outcomes.

## Conclusions

In conclusion, this hospital-based study demonstrated that nearly half of women with PCOS had concurrent metabolic syndrome, highlighting a substantial metabolic burden in this population. Obesity, central adiposity, and insulin resistance were identified as the strongest and most consistent predictors of metabolic syndrome, emphasizing their pivotal role in its pathogenesis. The findings reaffirm that PCOS is not merely a reproductive disorder but a systemic metabolic condition with long-term cardiovascular and endocrine implications. Early identification of metabolic abnormalities through routine screening, coupled with lifestyle modification, weight management, and targeted metabolic interventions, is crucial to prevent future complications. Given the high prevalence of obesity and sedentary behavior among Indian women, implementing culturally tailored preventive strategies at both clinical and community levels is essential to mitigate the growing dual epidemic of PCOS and metabolic syndrome.
